# Sporicidal performance induced by photocatalytic production of organic peroxide under visible light irradiation

**DOI:** 10.1038/srep33715

**Published:** 2016-09-26

**Authors:** Yuichi Yamaguchi, Takahito Shimodo, Noriyasu Chikamori, Sho Usuki, Yoshihiro Kanai, Takeshi Endo, Ken-ichi Katsumata, Chiaki Terashima, Masahiko Ikekita, Akira Fujishima, Tomonori Suzuki, Hideki Sakai, Kazuya Nakata

**Affiliations:** 1 Photocatalysis International Research Center, Research Institute for Science and Technology, Tokyo University of Science, 2641 Yamazaki, Noda, Chiba, 278-0022, Japan; 2 Department of Pure and Applied Chemistry, Faculty of Science and Technology, Tokyo University of Science, 2641 Yamazaki, Noda, Chiba, 278-0022, Japan; 3 Department of Applied Biological Science, Faculty of Science and Technology, Tokyo University of Science, 2641 Yamazaki, Noda, Chiba, 278-0022, Japan; 4 Research Institute for Science and Technology, Tokyo University of Science, 2641 Yamazaki, Noda, Chiba, 278-0022, Japan

## Abstract

Bacteria that cause serious food poisoning are known to sporulate under conditions of nutrient and water shortage. The resulting spores have much greater resistance to common sterilization methods, such as heating at 100 °C and exposure to various chemical agents. Because such bacteria cannot be inactivated with typical alcohol disinfectants, peroxyacetic acid (PAA) often is used, but PAA is a harmful agent that can seriously damage human health. Furthermore, concentrated hydrogen peroxide, which is also dangerous, must be used to prepare PAA. Thus, the development of a facile and safe sporicidal disinfectant is strongly required. In this study, we have developed an innovative sporicidal disinfection method that employs the combination of an aqueous ethanol solution, visible light irradiation, and a photocatalyst. We successfully produced a sporicidal disinfectant one hundred times as effective as commercially available PAA, while also resolving the hazards and odor problems associated with PAA. The method presented here can potentially be used as a replacement for the general disinfectants employed in the food and health industries.

World food culture is now diversified because the import and export of food is actively conducted all over the world. However, the internationalization of food has spread food poisoning diseases worldwide, resulting in large numbers of people suffering with diarrhea and vomiting annually; in worst-case scenarios, such infections can result in death. Additionally, massive group infection is an especially serious problem in the food and medical industries. Therefore, researchers around the world have sought to protect human health by actively investigating solutions to the food poisoning problem^1^,^2^,^3^,^4^,^5^,^6^,^7^,^8^. *Clostridium perfringens* is the feared pathogenic bacterium involved in serious food-borne disease outbreaks^9^,^10^,^11^,^12^,^13^,^14^. This organism causes life-threatening gas gangrene and mild enterotoxemia in humans^12^. A related bacterium, *Clostridium botulinum*, is considered a highly lethal pathogen, owing to its production of botulinum toxin, a highly potent poison^15^,^16^,^17^,^18^. This toxin can be lethal to humans at doses as low as 0.05 μg^19^, and has caused the death of many people worldwide who have ingested the microorganism. Under adverse conditions (low nutrient or limiting water), both *C. perfringens* and *C. botulinum* enter into a developmental pathway called sporulation. The resulting spores are a dormant cell type that possesses very high resistance to common sterilization methods, including heating at 100 °C and treatment with chemical agents. These resistance properties are attributed to the impermeability of a thick proteinaceous outer spore coat along with the stabilization of the cell contents within a spore core, which packages the DNA while containing high levels of pyridine-2,6-dicarboxylic acid (dipicolinic acid, DPA), and excluding water^20^,^21^,^22^,^23^,^24^. The spores cannot be inactivated with general alcohol disinfectants, necessitating the use of harsher agents such as hypochlorite disinfectants. However, hypochlorite is corrosive to metal and clothing owing to its strong basicity. Recently, peroxyacetic acid (PAA) has been adopted, but concentrated PAA is a harmful agent that can seriously damage human skin and respiratory systems, and this agent has a very strong odor^25^,^26^. Furthermore, concentrated hydrogen peroxide, which must be used to prepare the disinfectant, is itself dangerous. Thus, the development of a facile and safe sporicidal disinfectant is strongly required for applications in the food sector, the health industry, and the home. To resolve the problems described above, we developed a sporicidal disinfectant that uses a combination of an aqueous ethanol solution (commonly used as a disinfectant and known to be safe to human health), visible light irradiation, and a photocatalyst. Photocatalysis is a facile oxidative and reductive method whereby the photocatalyst compounds are activated only upon irradiation^27^,^28^,^29^,^30^,^31^,^32^,^33^,^34^,^35^,^36^. We expected that photocatalytic oxidative decomposition of ethanol would produce organic acids such as acetic acid and formic acid (known food components that are generally regarded as safe) along with hydrogen peroxide from the multi-electron reduction of atmospheric oxygen, such that the organic peroxide would endow the mixture with sporicidal activity.

To investigate the sporicidal performance of the method proposed in this study, we used spores of *Bacillus subtilis*, a model non-pathogenic spore former that is commonly used to evaluate techniques for the inactivation of bacterial spores. WO_3_ was used as a visible-light-driven photocatalyst. The band edge of the WO_3_ used in this work was estimated to be ca. 460 nm, as shown in Supplementary Figure 1, indicating a band gap of ca. 2.7 eV. The crystal structure of the WO_3_ was found to be monoclinic phase using X-ray diffraction (Supplementary Figure 2A). The particle size of the WO_3_ was estimated to be ca. 100 nm based on field emission scanning electron microscope (FE-SEM) observations (Supplementary Figure 3A). The surface area of the WO_3_ was calculated to be 7.6 m^2^ g^−1^ based on gas adsorption-desorption measurements.

We examined the survival rate of *B. subtilis* spores in the presence of WO_3_ suspended in aqueous ethanol disinfectant and irradiated with visible light. The dependence of the obtained sporicidal performance on the ethanol:water ratio was studied as shown in Fig. 1. A decrease in the survival rate of *B. subtilis* spores was first observed for the 6:4 (v/v) ethanol/water solution, with a ca. 2.5-log reduction observed after 24 h of irradiation. At ethanol:water ratios of 7:3, 8:2, and 9:1 (v/v), the spores were completely inactivated after 24, 9, and 12 h visible light irradiation, respectively. Furthermore, we investigated the sporicidal performance of ethanol:water solution at the indicated ratios (3:7, 6:4, 8:2, 9:1, v/v) without WO_3_. The reduction of survival rate of spore in aqueous ethanol solution was not observed as shown in Supplementary Figure 4.

These results indicated that the optimum sporicidal performance occurred at an ethanol/water ratio of 8:2 (v/v), and demonstrated that the inactivation of bacterial spores could be achieved by a facile and safe photocatalytic method.

To confirm the presence of organic peroxide, HPLC measurements were examined according to the method of Pinkernell *et al*.^37^,^38^. PAA oxidizes methyl p-tolyl sulfide (MTS) to methyl p-tolyl sulfoxide (MTSO) [equation 1].


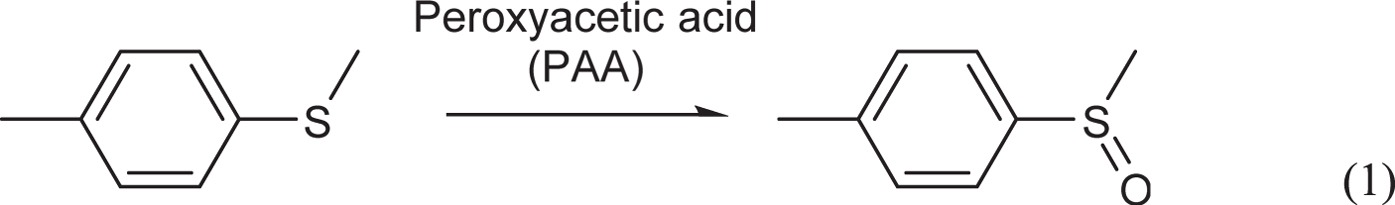


Therefore, the level of PAA can be quantified measuring the amount of MTSO generated. This method has the great advantage because the detection of PAA is not inhibited with the coexistence of hydrogen peroxide which typically oxidizes organic chemicals. A portion of the WO_3_ suspension after photocatalytic reaction was added to an MTS solution and the presence of MTSO was detected as shown in Fig. 2. The amount of organic peroxide produced gradually increased during the photocatalytic reaction, as shown in Fig. 3. The amount of organic peroxide produced after 12 h of visible light irradiation was estimated to be ca. 8.84 μmol (ca. 13.5 ppm), confirming the successful production of organic peroxide by photocatalytic oxidation of aqueous ethanol solution by WO_3_ illuminated with visible light. The organic peroxide was generated by an equilibrium reaction between hydrogen peroxide and organic acids [Equation 2]^26^,^39^,^40^.


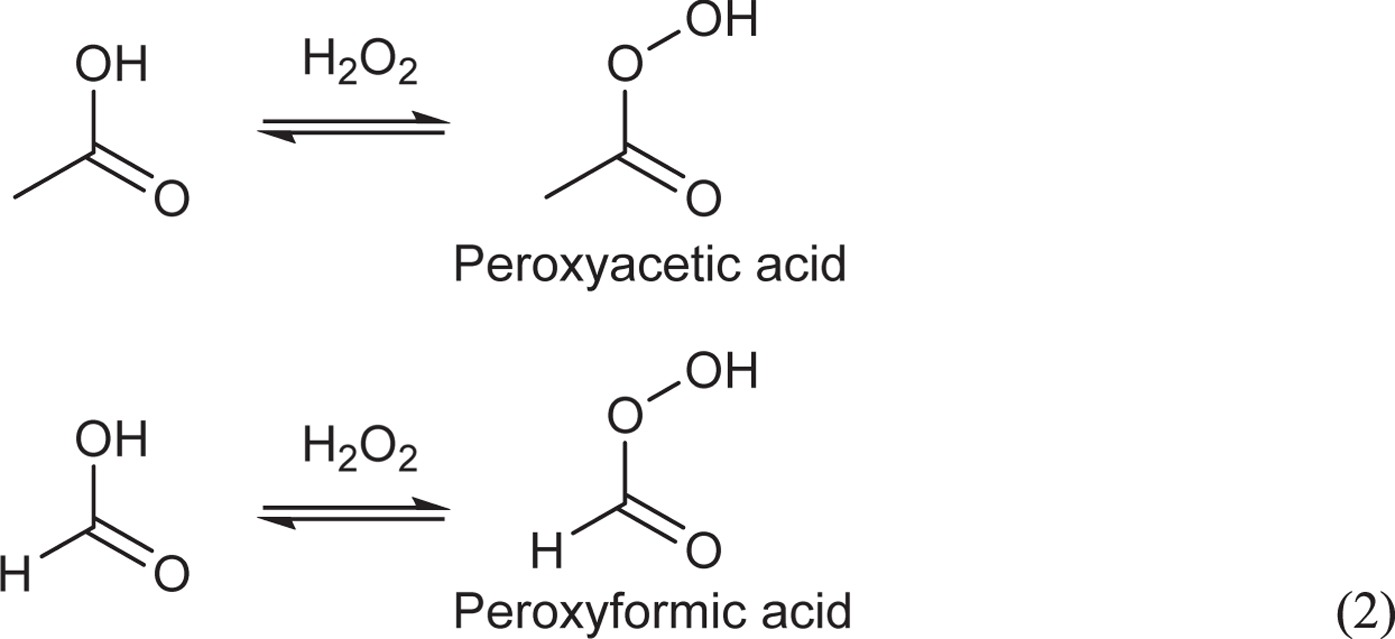


Therefore, we quantified the amounts of generated hydrogen peroxide, acetic acid, and formic acid using HPLC measurements. As can be seen in Fig. 3, the amounts produced increased with time of irradiation. After 12 h of irradiation, the amount of hydrogen peroxide, acetic acid, and formic acid generated in the reaction system approached 73.3 μmol (49.8 ppm), 107 μmol (129 ppm), and 64.5 μmol (59.4 ppm), respectively. The generation of hydrogen peroxide is known to proceed based on the multi-electron reduction of oxygen (O_2_ + 2H^+^ + 2e^−^ = H_2_O_2_, +0.68 V). The conduction band level of WO_3_ is negative enough (+0.5 V vs. NHE) for this multi-electron reduction to occur^41^,^42^,^43^. The photocatalytic oxidative decomposition of ethanol by photocatalysis proceeds with the formation of acetic acid and formic acid as intermediate products^44^,^45^. To promote the multi-electron reduction of oxygen, the presence of protons (H^+^) is of great significance. The equilibrium reaction between carboxylic acid and water proceeds as shown in equation 3, producing carboxylic species and H^+^.


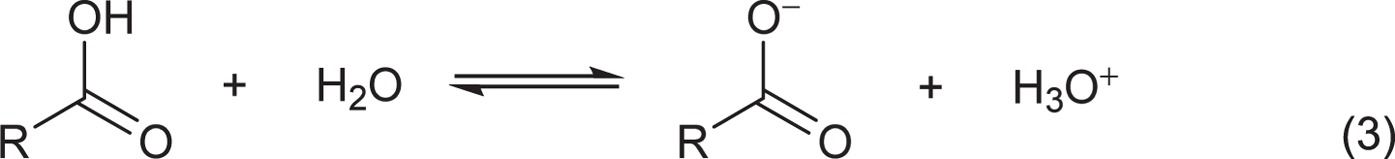


Thus, this equation indicates that the presence of water molecules is of great importance to the production of organic peroxide, and suggests that an optimum ethanol:water ratio exists. The method of Pinkernell *et al*. was used to estimate the amount of organic peroxide produced in the system. The cross reactivity between MTS and hydrogen peroxide also was investigated, in this case using a concentration of hydrogen peroxide (120 ppm) that was more than twice that produced by WO_3_ after 12 h of irradiation. As can be seen in Fig. 2, the hydrogen peroxide did not react with the MTS, indicating that the detected MTSO was derived from the presence of organic peroxide only.

Next, the sporicidal performance of a suspension of WO_3_ in 8:2 (v/v) ethanol:water after 12 h of visible light irradiation was compared with that of the commonly used, commercially available PAA disinfectant. Because the amount of generated organic peroxide was estimated (see above) to be ca. 13.5 ppm, 15 ppm PAA was used in the comparison. Figure 4 shows that the bacterial spores were completely inactivated (~6-log decrease in survival) in the solution treated with WO_3_ after 4 h of illumination. In contrast, 15 ppm commercial PAA disinfectant solution did not provide appreciable killing (<0.5-log decrease) in the same interval. Testing at higher PAA concentrations revealed that the WO_3_ system exhibited a sporicidal performance equivalent to 1500 ppm PAA, indicating that our method has a sporicidal effect 100 times that of PAA. Additionally, we note that the suspension treated with WO_3_ contained not only acetic acid but also formic acid. We therefore presume that peroxyformic acid, which is a stronger oxidant than PAA, was produced in the presence of WO_3_. The generation of peroxyformic acid may account for the remarkable sporicidal performance of the suspension treated with WO_3_ compared to that observed in the suspension treated with the commercial PAA disinfectant.

The presence of hydrogen peroxide is critical to the generation of organic peroxide. The conduction level of a photocatalyst is closely related to generation of hydrogen peroxide. To demonstrate the importance of the conduction level, we evaluated the survival rate of *B. subtilis* spores in a system containing TiO_2_, which is (globally) the most widely used and applied photocatalyst. It is well known that although TiO_2_ exhibits high photocatalytic activity, its conduction band level is much more negative than the potential of the multi-electron reduction of oxygen. As a result, the single-electron reduction of oxygen (O_2_ + e^−^ = O_2^−^_, −0.284 V; O_2_ + H^ +^ + e^−^ = HO_2_, −0.046 V) preferentially proceeds over TiO_2_ instead^46^,^47^,^48^. Also, it was reported that O_2^−^_ and HO_2_ can transfer to H_2_O_2_ through some subsequent reactions^48^,^49^,^50^. However, TiO_2_ should rapidly consume hydrogen peroxide producing during irradiation as shown in Supplementary Figure 5, which suppressed increasing the concentration of hydrogen peroxide. Hence, it seems that organic peroxide cannot be generated via TiO_2_. The band edge of the TiO_2_ used in this work was observed at ca. 400 nm, as shown in Supplementary Figure 1, indicating an estimated band gap of 3.1 eV. The crystal structure of the TiO_2_ was a mixture of anatase and rutile phases (Supplementary Figure 2B), and the particle size was estimated to be ca. 20 nm (Supplementary Figure 3B). The surface area of the TiO_2_ was found to be 54 m^2^ g^−1^, larger than that of the WO_3_. Supplementary Figure 6 shows that the *B. subtilis* spores were not inactivated in aqueous ethanol solution upon illumination with either UV or visible (λ > 420 nm) light, indicating that TiO_2_ is not suitable for the photocatalytic inactivation of bacterial spores. Next, the quantitative amount of organic peroxide, hydrogen peroxide, acetic acid, and formic acid produced in the presence of TiO_2_ in 8:2 (v/v) ethanol/water under UV light irradiation was investigated. As shown in Fig. 2, no MTSO peak detected, indicating that the TiO_2_ did not produce organic peroxide. As shown in Supplementary Figure 7, the formation of acetic acid and formic acid was confirmed, indicating that the photocatalytic oxidative reaction proceeded. In contrast, the production of hydrogen peroxide was not observed, in accordance with the difficulty of the multi-electron reduction of oxygen over TiO_2_. These results revealed that WO_3_ is a more suitable visible-light-driven photocatalyst for the inactivation of bacterial spores than TiO_2_, and suggests that the presence of hydrogen peroxide is key to the production of organic peroxide. As shown in Fig. 5, the valence band levels of TiO_2_ and WO_3_ have similar positions because band levels consist of O2p orbitals, which enable the photocatalytic oxidative decomposition of ethanol to proceed. However, the conduction band level of TiO_2_ is much more negative than that of WO_3_, which causes the single-electron reduction of oxygen to preferentially occur. This difference explains why illuminated TiO_2_ did not produce hydrogen peroxide, precluding the formation of organic peroxide in the presence of this photocatalyst.

The efficient sporicidal performance obtained with a low concentration of organic peroxide in this study resolves the odor problem and danger encountered with traditional sporicidal disinfectants. Hence, the present method is superior not only for the food and health industries, but also for use by the general public at home. This result is of great significance in terms of the production of an efficient sporicidal disinfectant from a safe chemical agent, aqueous ethanol, using not UV but visible light illumination. One of the next goal of this sporicidal sterilization method is more rapid inactivation of bacterial spores. If it could be achieved, this novel method is expected to become prevalent owing to the possibility that the inactivation of bacterial spores may be realized simply by squirting an aqueous ethanol solution onto substrates coated with photocatalyst under visible light.

## Methods

### Materials

TiO_2_ and WO_3_ were purchased from Evonik and Sigma-Aldrich, respectively, and used as received without any purification.

### Characterization

Diffuse reflectance spectra were obtained using a UV-VIS spectrometer (JASCO V-670) and were converted from reflection to absorbance using the Kubelka-Munk formula. Crystal structures were determined using X-ray diffraction (XRD; RIGAKU Ultima IV) with CuKα radiation. Particle morphologies were observed using a field emission scanning electron microscope (FE-SEM; JEOL-7600F) accelerated at 15 kV. Samples for FE-SEM were sputtered with 10 nm Au metal. Surface areas were determined by the Brunauer-Emmett-Teller (BET) method using a gas adsorption and desorption analyzer (MicrotracBEL; BELSORP-max) with nitrogen gas at 77 K.

### Preparation of spores

*Bacillus subtilis* spores were used for evaluation of sporicidal activity. Vegetative cells of *B. subtilis* (IAM12118^T^) were grown and sporulated by incubation on nutrient agar (NA) medium for 10 days at 37 °C. The spores were collected with 10 mL ultra-pure water. Lysozyme (100 mg) was added to the suspension to remove remaining vegetative bacterial cell wall. The suspension then was incubated for 30 min at 37 °C and washed three times with 10 mL ultra-pure water with harvesting by centrifugation at 3500 rpm. The formation of *B. subtilis* spores was confirmed by the Wirtz method using malachite green and safranin stains. Additionally, no decrease in the survival rate of *B. subtilis* spores was observed in ethanol/water (70:30, v/v), suggesting the formation of spores (i.e., ethanol-resistant cells).

### Evaluation of photocatalytic inactivation of spores

The density of spores was adjusted to 2.0 × 10^6^ CFU/mL. Photocatalyst (25 mg) was added to glass containers containing 50 mL of spores suspended in the ethanol/water solution. Photo-irradiation was carried out using a black light (2.0 mW cm^−2^) for UV irradiation and a Xe lamp (110 mW cm^−2^) with L-42 cut-off filter (HOYA, λ < 420 nm) under magnetic stirring for visible light irradiation. After illumination for the indicated time interval, dilutions of the suspension were plated to NA, and the plates were incubated for 48 h at 37 °C. The spore survival rate was determined using the colony counting method.

### Comparison of sporicidal performance of commercially available PAA (15, 150, 500, 1000, and 1500 ppm) and suspension treated with WO_3_ in ethanol:water (8:2, v/v) after 12 h visible light irradiation

The concentration of bacterial spores was adjusted to 2.0 × 10^6^ CFU/mL. Each concentration of PAA was obtained by diluting commercially available 6% PAA (Sigma-Aldrich) with ultra-pure water. Spore suspension was added to the solution of PAA or the suspension of WO_3_ in ethanol:water (8:2, v/v) after 12 h of visible light irradiation. After the irradiation, the WO_3_-treated suspension was centrifuged at 10000 rpm for 10 min to remove the WO_3_ powder. The sporicidal performance of the treatments was evaluated as above.

### Determination of organic acid, hydrogen peroxide, and organic peroxide levels

Levels of formic acid and acetic acid were quantified using a HPLC (Shimadzu; LC-20AD) with a UV detector (Shimadzu; SPD-20A) at a wavelength of 210 nm. The HPLC conditions were as follows: column 300 × 7.8 mm (Phenomenex; Rezex ROA-Organic Acid), column temperature 60 °C, mobile phase 0.005 N sulfuric acid, flow rate 0.5 mL/min. The photocatalytically treated samples were diluted 10-fold with ultra-pure water before loading to prevent deterioration of the column.

Hydrogen peroxide was detected using a HPLC (Hitachi; Chromaster) with a UV detector (Hitachi; Chromaster 5410) at a wavelength of 210 nm. The HPLC conditions were as follows: column 250 × 4.6 mm (Hitachi; LACHROM 2 C18), column temperature 25 °C, mobile phase 0.1% phosphoric acid, flow rate 1.0 mL/min.

Organic peroxide was detected using a HPLC (Hitachi; Chromaster) with a UV detector (Hitachi; Chromaster 5410) at a wavelength of 254 nm. The HPLC conditions were as follows: column LACHROM 2 C18, column temperature 40 °C, mobile phase 75% ethanol/25% water (v/v), flow rate 1.0 mL/min. The photocatalytically treated samples (0.5 mL) were mixed with MTS ethanol solution (50 ppm, 0.5 mL) at 25 °C for 2 min. The concentration of organic peroxide was quantified using the area of the MTSO peak.

## Additional Information

**How to cite this article**: Yamaguchi, Y. *et al*. Sporicidal performance induced by photocatalytic production of organic peroxide under visible light irradiation. *Sci. Rep.* **6**, 33715; doi: 10.1038/srep33715 (2016).

## Supplementary Material

Supplementary Information

## Figures and Tables

**Figure 1:**
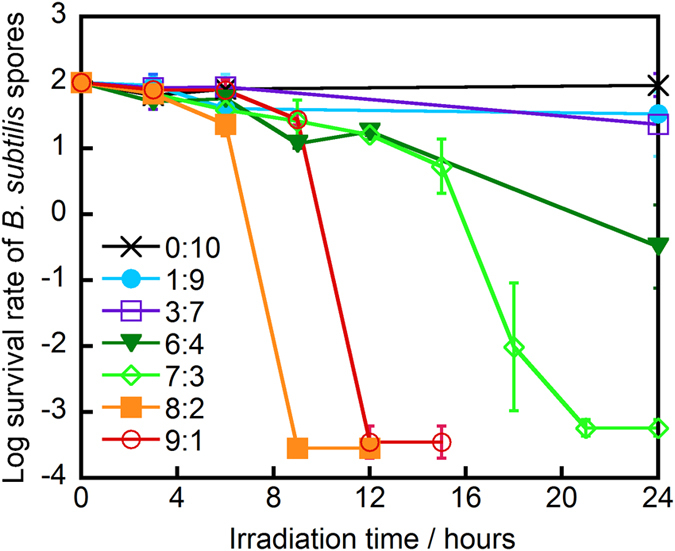
Survival rate of *B. subtilis* spores in the presence of WO_3_ suspended in ethanol:water solution at the indicated ratios ((a) 0:10; (b) 1:9; (c) 3:7; (d) 6:4; (e) 7:3; (f) 8:2; (g) 9:1, v/v) and illuminated with visible light for the indicated time. Photocatalyst: 25 mg, light source: Xe lamp (vis) with L-42 filter (λ > 420 nm), liquid-phase volume: 50 mL, density of *B. subtilis* spores: 2.0 × 10^6^ CFU/mL.

**Figure 2:**
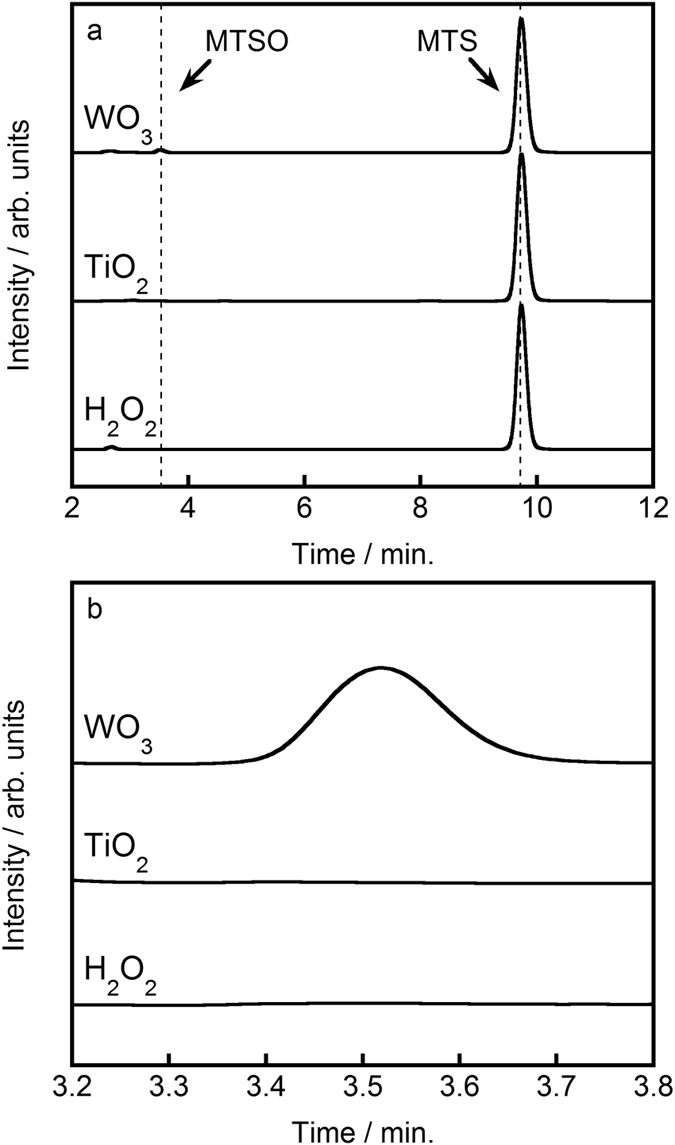
HPLC chromatograms of MTSO and MTS. (**a**) WO_3_ in ethanol:water solution (8:2, v/v) and illuminated with visible light for 12 h, TiO_2_ in ethanol:water solution (8:2, v/v) and illuminated with UV light for 24 h, and 120 ppm hydrogen peroxide. (**b**) Expanded view of the time interval from 3.2 to 3.8 min in panel (**a**).

**Figure 3:**
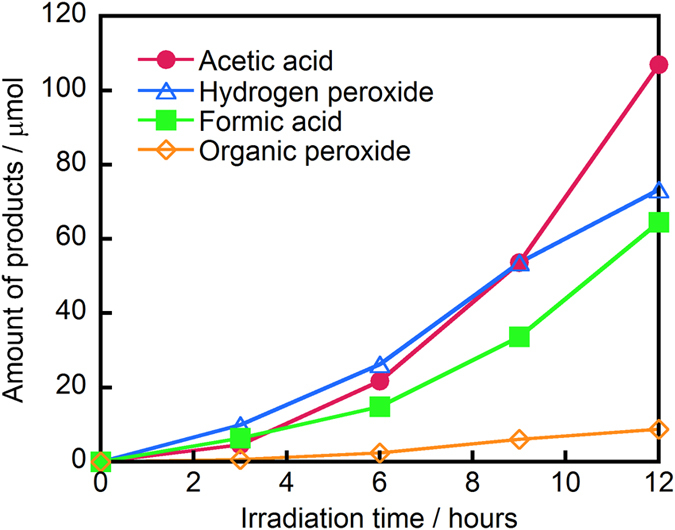
Time-dependence of amount of organic peroxide, hydrogen peroxide, acetic acid, and formic acid produced by WO_3_ suspended in ethanol:water solution (8:2, v/v) illuminated with visible light (λ > 420 nm) for the indicated time.

**Figure 4:**
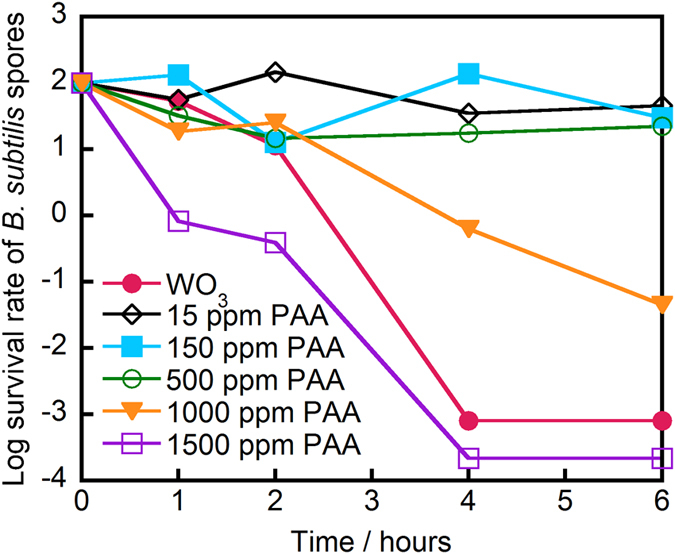
Survival rate of *B. subtilis* spores for the indicated time under dark conditions after the treatment, either of commercially available peracetic acid (PAA) solution at various concentrations (15, 150, 500, 1000, or 1500 ppm) or of WO_3_ suspended in ethanol:water solution (8:2, v/v) after 12 h of visible light irradiation. Photocatalyst: 25 mg, light source: Xe lamp (vis) with L-42 filter (λ > 420 nm), liquid-phase volume: 50 mL, density of *B. subtilis* spores: 2.0 × 10^6^ CFU/mL.

**Figure 5:**
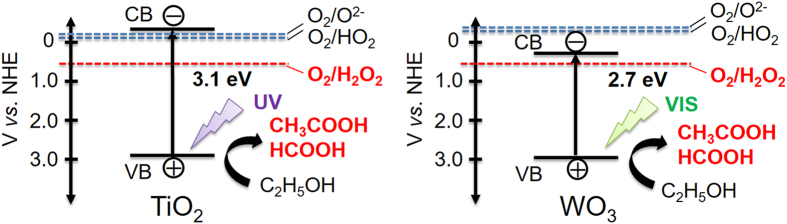
Relationship between the potential of oxygen reduction and the band structures of TiO_2_ and WO_3_.
