# Supplementary material for: Sporicidal performance induced by photocatalytic production of organic peroxide under visible light irradiation
Source: Sci Rep. 2016 Sep 26;6:33715. doi: 10.1038/srep33715 (PMC5036025; doi:10.1038/srep33715)
Supplement: Supplementary Information [file srep33715-s1.doc]

**Supplementary Information**

**Sporicidal Performance Induced by Photocatalytic Production of Organic Peroxide under Visible Light Irradiation**

Yuichi Yamaguchi, Takahito Shimodo, Noriyasu Chikamori, Sho Usuki, Yoshihiro Kanai, Takeshi Endo, Ken-ichi Katsumata, Chiaki Terashima, Masahiko Ikekita, Akira Fujishima, Tomonori Suzuki, Hideki Sakai & Kazuya Nakata

**
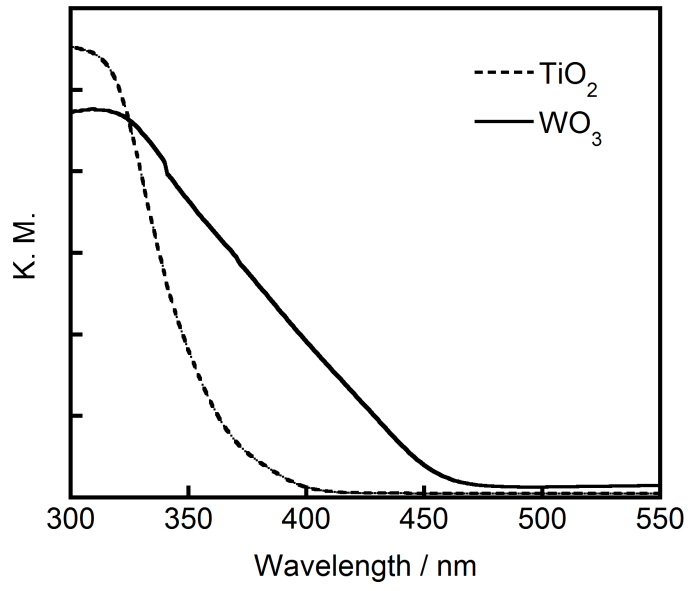
**

**Supplementary Figure 1.** Diffuse reflectance spectra of TiO_2_ and WO_3_.

**
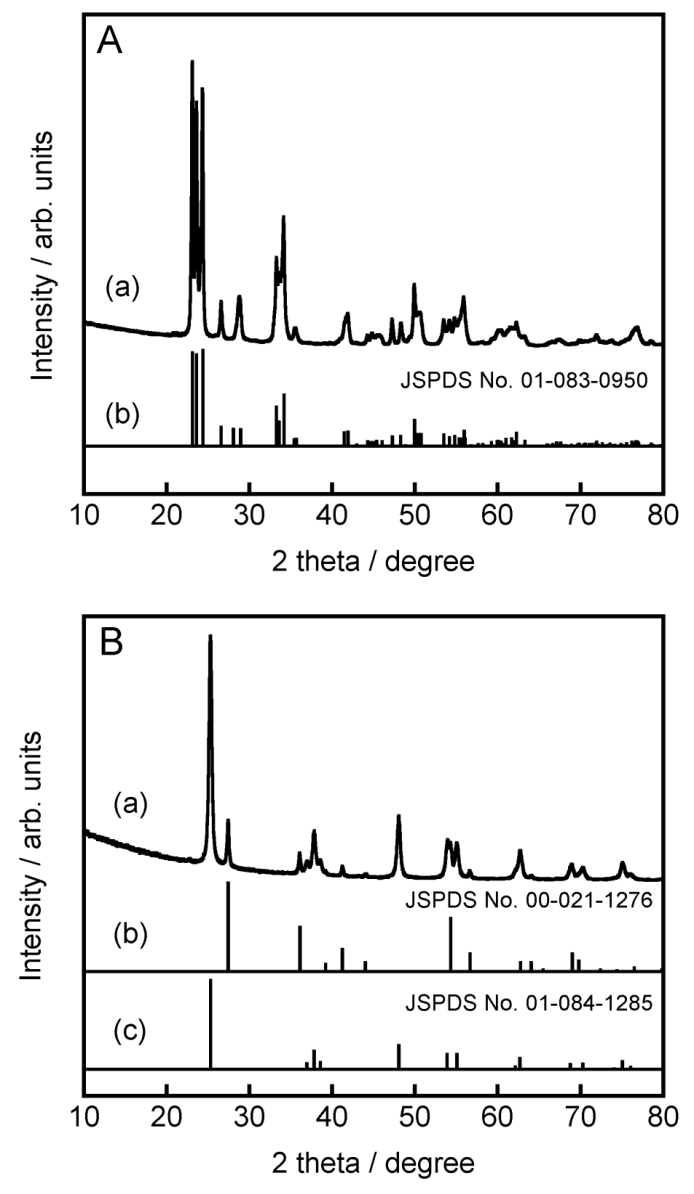
**

**Supplementary Figure 2.** X-ray diffraction patterns of (A) WO_3_, (a) WO_3_ sample, (b) monoclinic WO_3_ (JSPDS No. 01-083-0950), (B) TiO_2_ (P-25), (a) TiO_2_ sample, (b) rutile TiO_2_ (JSPDS No. 00-021-1276), (c) anatase TiO_2_ (JSPDS No. 01-084-1285).

**
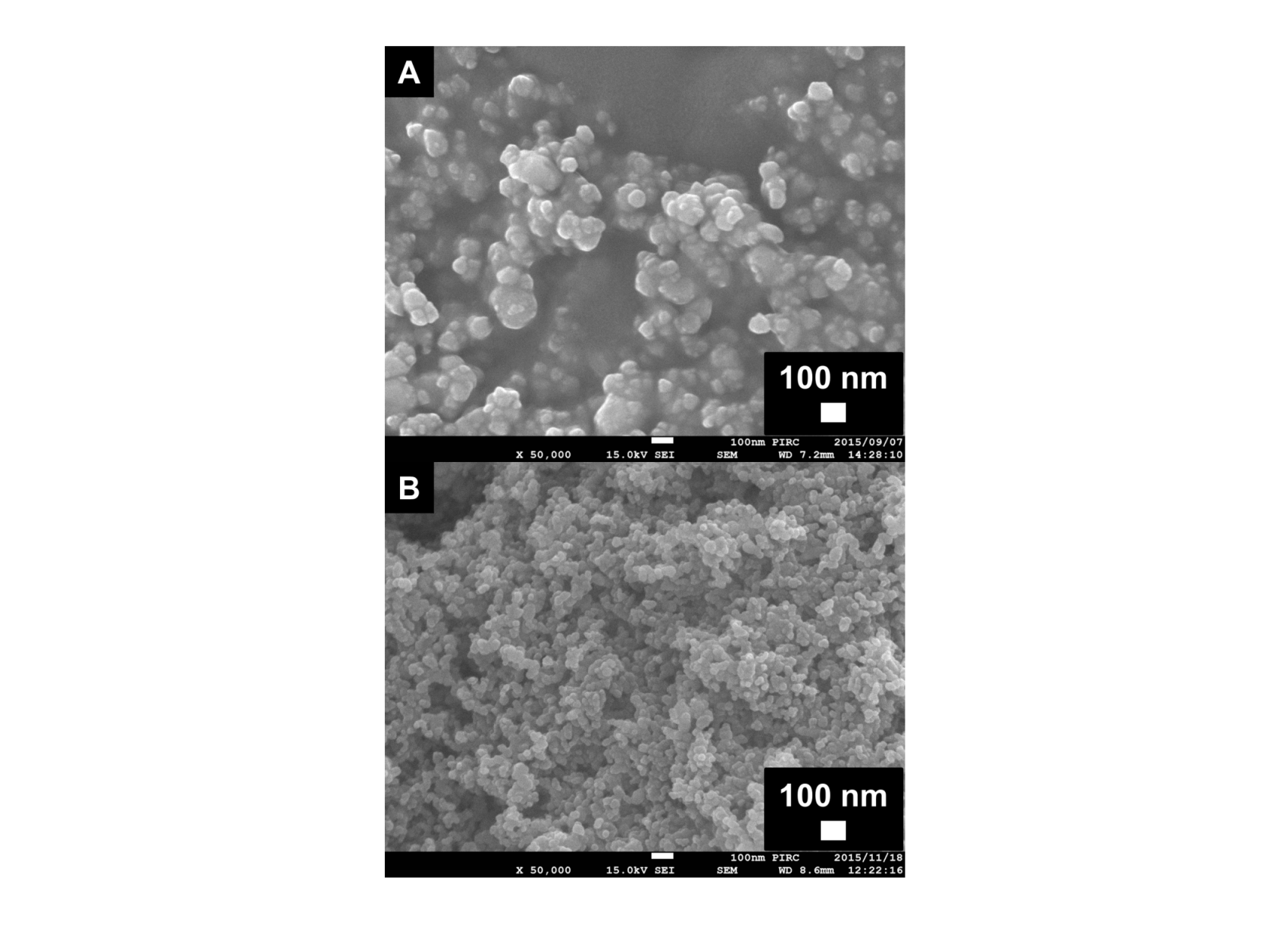
**

**Supplementary Figure 3.** Field emission scanning electron microscope images of (A) TiO_2_ and (B) WO_3_.


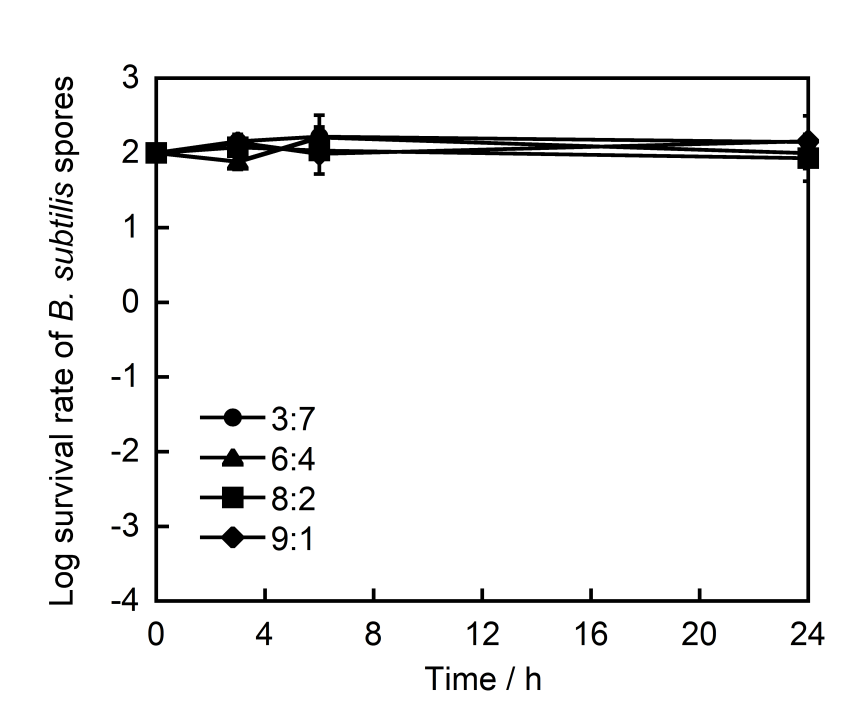


**Supplementary Figure 4.** Survival rate of *B. subtilis* spores in ethanol:water solution at the indicated ratios (3:7, 6:4, 8:2, 9:1, v/v) without WO_3_.

**
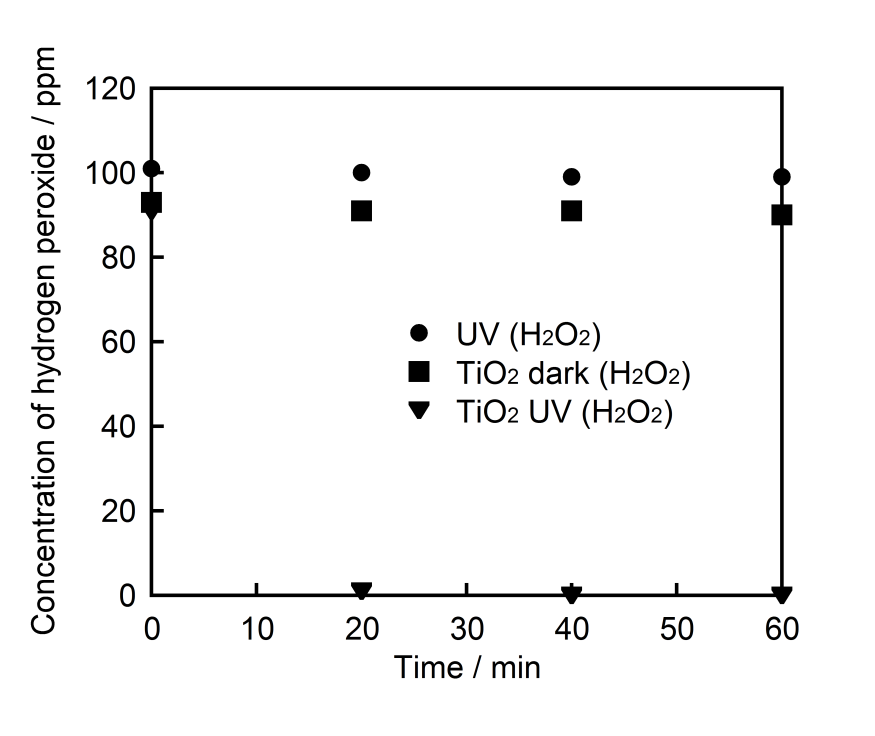
**

**Supplementary Figure 5.** Photocatalytic decomposition of hydrogen peroxide (100 ppm) with TiO_2_ under UV light irradiation. Photocatalyst: 15 mg, light source: black light (UV; 1 mW cm^-2^), liquid-phase volume: 30 mL.

**
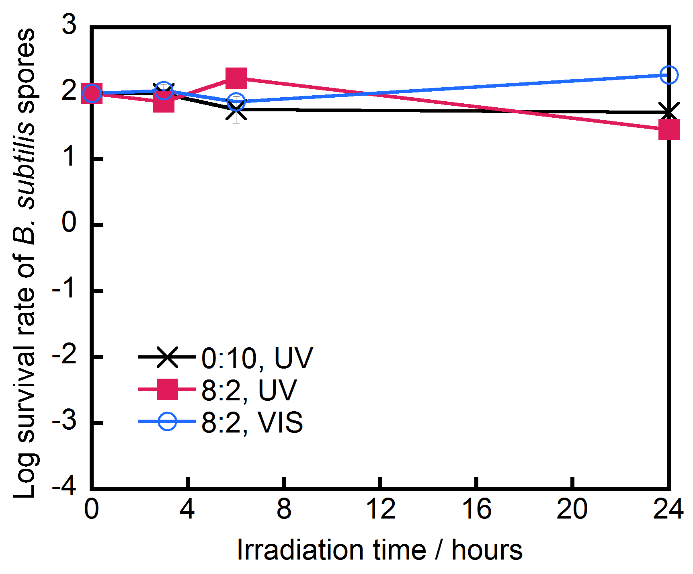
**

**Supplementary Figure 6.** Survival rate of *B. subtilis* spores in the presence either of TiO_2_ suspended in ethanol:water solution at indicated ratio (0:10, 8:2; v/v) and illuminated with UV light for the indicated time, or of TiO_2_ suspended in ethanol:water solution (8:2, v/v) and illuminated with visible light for the indicated time. Photocatalyst: 25 mg, light source: black light (UV), Xe lamp (VIS) with L-42 filter (λ > 420 nm), liquid-phase volume: 50 mL, density of *B. subtilis* spores: 2.0 × 10^6^ CFU/mL.

**
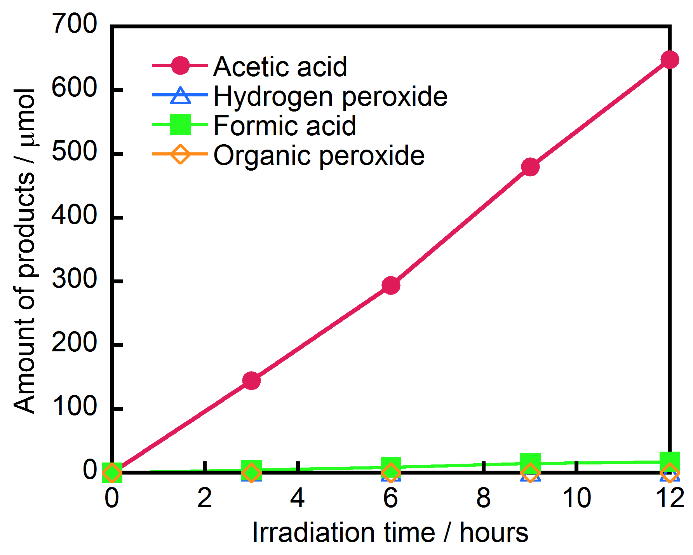
**

**Supplementary Figure 7.** Time-dependence of amount of organic peroxide, hydrogen peroxide, acetic acid, and formic acid produced by TiO_2_ suspended in ethanol:water solution (8:2, v/v) and illuminated with UV light for the indicated time.
